# Supplementary material for: Microbial Community Composition of Explosive-Contaminated Soils: A Metataxonomic Analysis
Source: Microorganisms. 2025 Feb 19;13(2):453. doi: 10.3390/microorganisms13020453 (PMC11858405; doi:10.3390/microorganisms13020453)
Supplement: Supplementary file 1 [file microorganisms-13-00453-s001.zip › microorganisms-3392879-supplementary.pdf]

**Supplementary Table S1.** Shapiro-Wilk normality test of alpha diversity data. p-value > 0.05 suggests that the data follows a normal distribution.

| Site | Type | p-value | | | |
|---|---|---|---|---|---|
| | | Observed | Shannon | Simpson | Chao 1 |
| Bacteria | Soil | 0.1327 | 0.1003 | 0.6295 | 0.000003914 |
| Bacteria | Root | 0.9795 | 0.4379 | 0.1274 | 0.1275 |
| Fungi | Soil | 0.1144 | 0.2122 | 0.01878 | 4.278e-06 |
| Fungi | Root | 0.03569 | 0.02782 | 0.6277 | 0.004117 |

A

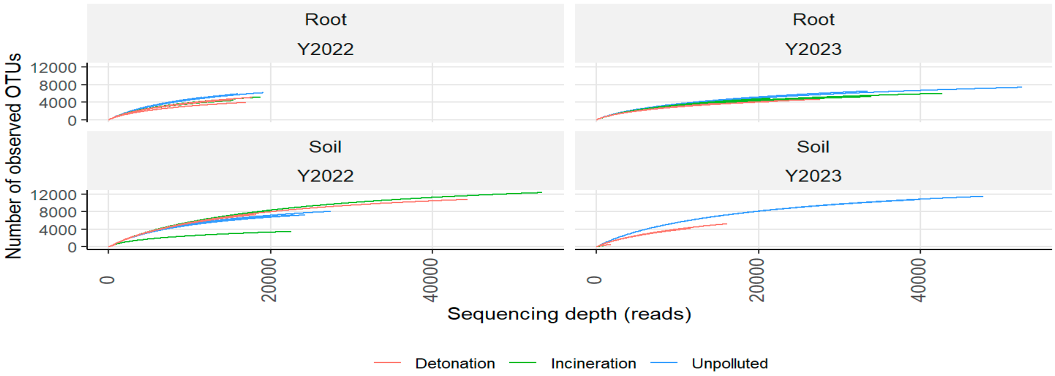


B

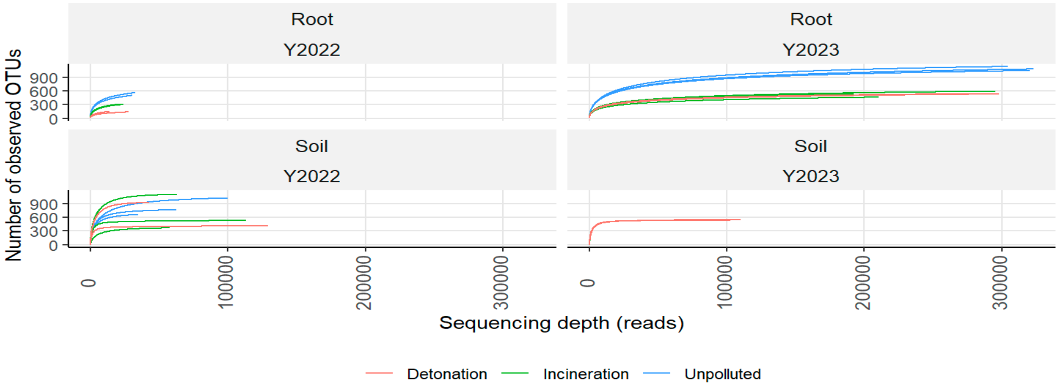


**Supplementary Figure S1.** Rarefaction curves A. Bacteria, B. Fungi.
